# Exploring the development of coach-parent relationships in organized competitive youth team sports

**DOI:** 10.1371/journal.pone.0333559

**Published:** 2025-12-08

**Authors:** Sina Azimi, Jordan Sutcliffe, Katherine Tamminen

**Affiliations:** 1 Faculty of Kinesiology and Physical Education, University of Toronto, Canada; 2 Department of Military Psychology and Leadership, Royal Military College of Canada, Kingston, Canada; Universidade Federal de Goias, BRAZIL

## Abstract

The purpose of this research was to gain a better understanding of coach-parent relationships in organized competitive youth team sports. Two qualitative studies were conducted with distinct samples from youth sports teams (U10-U15) within a large urban center in Ontario, Canada. Study 1 explored perceptions of key relationship-shaping factors among coaches (*n* = 21; 5 females, 16 males; age range: 22–64 years) and parents (*n* = 20; 6 females, 14 males; age range: 40–63 years). Identified factors included behavioural expectations, communication practices, trust, and pressures associated with professionalization. Study 2 (*n* = 20; 10 parents [9 females, 1 male] and 10 coaches [2 females, 8 males]) utilized grounded theory to propose a three-stage developmental model: (a) Introduction and discovery, (b) Curiosity, doubt, or stability, and (c) Engagement or separation. This model provides a clear framework illustrating how coach-parent interactions evolve, stabilize, or deteriorate, influenced by broader sociocultural and structural factors such as generational differences, organizational policies, and sport professionalization. Results emphasize the strategic importance for coaches and sport organizations in managing parent relationships and outline practical implications for enhancing these interactions. This research addresses an important gap by offering a theoretically grounded, developmental perspective on coach-parent dynamics in youth sport.

## Introduction

Social agents such as parents and coaches can greatly impact youth athletes’ motivation and experiences in sport by providing autonomy support, promote effort/mastery, and display positive emotional responses [1]. Parents play a vital role in their children’s sport-related socialization, ensuring positive outcomes by offering support, praise, and using ‘teachable moments’ for their child’s development [2]. Parental communication before, during, and after sporting events is another important aspect of parental involvement that has been studied in youth sport [3,4,5]. More specifically, parental communication has the potential to create either enjoyable or uncomfortable experiences for youth athletes [4,5]. Recent research examining parent and child car-ride interactions before and after sport competitions demonstrated that parents provided more praise about their child’s performance than athletes did themselves [6]. Nonetheless, recent research continues to highlight the detrimental impact of parental pressure [i.e., demonstrated by parents overstepping boundaries and holding unrealistic expectations] on athletes’ experiences [7]. In summary, parents are one of the primary actors in youth sport and can positively or negatively influence athletes’ experiences through their values, attitudes, and communication.

In addition to parents, coaches greatly influence athletes’ development and enjoyment. Coaches shape young athletes’ experiences and can enhance pro-social behaviours while discouraging anti-social behaviours [8,9], and the motivational climate created by coaches and their associated behaviours and leadership influence athletes’ motivation, well-being, and performance [10]. A positive coach-athlete relationship is associated with athletes’ self-competence and motivation to participate in sport [10]. Furthermore, interactions with coaches can serve as a protective influence or a risk factor for athlete burnout [11] and can compensate in protecting athletes’ motivation in instances where parents provide low autonomy support for athletes [12]. Hence, the coach-athlete relationship can significantly impact athletes’ self-competence and motivation to participate in sport.

It is also apparent that the relationship between coaches and parents can influence athletes’ experiences in sport. The coach-parent relationship can be conceptualized as recurring activities and communications between parents and coaches, which focus mainly on the shared task of supporting the athlete [13]. In a recent scoping review of coach-parent relationships and interactions in youth sport settings [14], a comprehensive overview of effective and ineffective practices was articulated. Effective practices emphasize building meaningful partnerships through regular social interaction and communication, recognizing parents as integral members of the team, and fostering open, honest, and respectful dialogue between coaches, parents, and athletes [15,16]. This approach reflects the growing consensus that coach-parent relationships can influence both the developmental outcomes of athletes and the success of youth sport programs [17]. Ineffective practices, such as verbal or physical abuse, lack of communication, and insufficient trust between coaches and parents, were found to negatively impact athletes’ experiences and retention [18,19]. Additional challenges in the coach-parent relationship include a lack of time for meaningful engagement, poor autonomy for coaches, and minimal training on fostering positive coach-parent dynamics [20]. Importantly, researchers suggest that simply avoiding these ineffective behaviors is insufficient for ensuring productive relationships, and instead, support programs are recommended to help coaches and parents reflect on their roles and align their practices to build productive relationships and support positive athlete outcomes [21].

Although the review by Santos et al. [14] provides a helpful synthesis of the field, the coach-parent relationship may look different depending on the sport and level of competition. For example, in an examination of the coach-parent relationship in competitive figure skating, parents described their experiences of the relationship as fluctuating from negative and distant to positive and enjoyable [13]. Participants also variously described their relationship in relation to the athlete as collaborative, coach-athlete centric, and contractual [13]. Furthermore, researchers have explored the coach-parent relationship in competitive youth hockey [16], highlighting the importance of mutual trust and respect between parents and coaches [16]. In addition, shared experiences, parents’ recognition of their own inexperience, and parents’ level of involvement were shown to influence the coach-parent relationship [16]. Likewise, coaches initiating frequent and honest interactions with parents, while setting communication boundaries to prevent or manage potential conflicts were recommended strategies to optimize the coach-parent relationship [16]. In their recent research, O’Donnell et al. [22] explored the coach-parent relationship through a sociocultural lens across a variety of team and individual sports [e.g., badminton, netball, running soccer, basketball, etc]. Findings from this study suggest that the coach-parent relationship is developed and formed by broader aspects of society and culture, and beyond social exchanges with one another [22]. Furthermore, these findings reveal that formal and informal credentials, intermediary roles, sharing values, goals, and expectations, and managing relational boundaries are socially significant for the development of the coach-parent relationship [22]. These findings demonstrate the complexity and the fluidity of the coach-parent relationship in competitive youth sport settings.

While a number of studies have explored the nature of coach-parent relationships, there is also evidence that parent- and coach-related stressors influence the quality of the coach-parent relationship. For example, some of the parent-related stressors perceived by coaches include demands for time, unrealistic expectations and pressure, varying levels of involvement and engagement, over-emphasis on winning, lack of respect and trust, and interfering with coaching decisions [23]. Parents also expressed distinct expectations for quality coaching, emphasizing not only the need for coaches to possess technical skills and knowledge but also to develop players holistically, focusing on personal growth and resilience. However, many parents identified limitations of volunteer-based settings, recognizing that coaches often lack the expertise or time to meet these expectations [24]. Similarly, a vignette study by Sutcliffe et al. [25] reported that youth parents prefer their children’s coaches to have characteristics that exemplify competence more than warmth. On the other hand, coach-related stressors [i.e., lack of communication, showing favouritism] have shown to count for 15% of parents’ competitive stressors related to their child’s sport participation [26]. Consequently, parents may ultimately choose to remove their child from that training environment, which may negatively impact the child’s social relationships with their teammates and peers. Thus, to create an optimal environment for youth athletes, and to improve parents’ and coaches’ experiences, it is important that parent and coaches work together to co-produce quality youth sport experiences.

In order to support youth athletes, equitable interactions are built through mutual trust and relationship-building, with both parents and coaches adjusting their roles based on the athlete’s progress and circumstances [15]. However, a key element to consider in these relationships is the underlying power dynamic between parents and coaches. While parents often control access to sport programs, coaches must navigate these dynamics carefully to maintain collaborative partnerships. Coaches must set boundaries to protect their role and avoid being undermined by over-involved parents, while also balancing their need to maintain positive relationships [15].

### Remaining gaps in the literature

Despite the existing research on the topic of coach-parent relationship, certain limitations exist that warrant further discussion. First, Preston et al.’s [16] investigation of the coach-parent relationship focused on one male Canadian minor hockey team; thereby limiting the findings to only one context in the youth sport environment, and their study did not seek to explain the development of the coach-parent relationship. Similarly, participants in Harwood et al. [27] were asked to discuss their positive or negative experiences of the coach-parent relationship; however, limited information was provided about how those experiences developed over time. A focused examination of the development of coach-parent relationships would help to advance this gap in the literature by documenting the ways that their relationship may change and evolve over time. Second, despite demonstrating that the coach-parent relationship can be experienced and perceived differently by parents and coaches, Wall et al. [13]’s empirical work only included mothers and focused on an individual/co-acting sport. Thus, additional research examining coach-parent relationships in team sport contexts is warranted. Finally, coach-parent relationship research has relied on primarily quantitative approaches, thus limiting a richer understanding of how coach-parent relationships develop [18], a lack of clarity in relation to the level of sport from which participants were recruited from [22], and inadequate information about the coach-parent relationship and its development in team environments [18,28]. Therefore, while prior studies have outlined important relational stressors and contextual factors in coach-parent interactions, there remains a limited understanding of how these relationships develop over time, particularly in competitive team sport settings. Much of the existing research focuses on static descriptions, individual sports, or single-sided perspectives (e.g., only parents or only coaches), and relies heavily on quantitative methods. These limitations highlight the need for inductive, developmentally focused research that captures the dynamic and reciprocal nature of coach-parent relationships in youth team sport.

### The current study

The aims of this research were twofold: (a) to explore parents’ and coaches’ perceptions of the factors that influence the coach-parent relationship in organized competitive youth team sports, and (b) to develop a conceptual model that explains the developmental trajectory of the coach-parent relationship in organized competitive youth team sports. Despite growing attention to coach-parent dynamics, existing literature remains limited in three ways: a lack of longitudinal or developmental focus, a reliance on individual or co-acting sport contexts, and a predominant use of quantitative methods that may overlook the nuances of lived experience. Additionally, no comprehensive theoretical framework currently exists to explain how coach-parent relationships evolve over time in youth team sport settings. To address these gaps, an initial qualitative exploration of parents’ and coaches’ experiences of the coach-parent relationship was undertaken, followed by a grounded theory [29] study to develop a conceptual model to explain the development of the coach-parent relationship. Study one and two were conducted with two distinct samples of participants.

### Philosophical assumptions

The researcher adopted a constructivist paradigm consisting of a relativist ontology and a subjectivist and transactional epistemology [30]. The main aim of research conducted within a constructivist paradigm is to understand and interpret meanings of phenomena that are obtained from construction/reconstruction of lived experiences [30]. Adopting a relativist ontology means that it is understood that coaches and parents may form different interpretations and mental constructions about a phenomenon, and that those interpretations may have been influenced by their lived experiences and previous social interactions. Similarly, within this research, assuming a subjectivist and transactional epistemology means that knowledge and findings are created as a result of multiple interactions between the researcher and participants, and the researcher’s interpretations of those interactions.

The purpose of Study One was to explore parents’ and coaches’ perceptions of the factors that influence the coach-parent relationship in competitive youth team sports. This study addressed the following research questions: [a] How do parents and coaches involved in competitive youth sports perceive and experience their relationships with one another? and [b] What factors do parents and coaches perceive to influence their relationship with one another?

## Methods

### Participant recruitment and data collection

This research was approved by the University of Toronto Research Ethics Board [REB #39710] and informed written consent was obtained from all participants prior to data collection. Upon receiving ethics approval from the researchers’ institutional research ethics board, the first author recruited parents and coaches of youth competitive sports teams (U10-U15) within a large urban center in Ontario, Canada. The recruitment process began through purposeful and snowball sampling [31] of participants who may provide the best data for addressing the research questions. Snowball sampling was used to recruit additional participants through existing study subjects [31]. The recruitment period for both Study One and Study Two began 01/10/2021 and ended 30/09/2022.

Club administrators and coaches from various team sports (e.g., soccer, hockey, basketball, and baseball) with public contact information were contacted via phone and e-mail and asked to send information about the study to parents and other coaches in their organization. Information about the study was also posted on social media platforms (i.e., Twitter) where club administrators, coaches, and parents could view and share this information. Parents and coaches could contact the researcher via email to express their interest in participating in the study. Parents and coaches were provided with information letters and provided written consent prior to participating in the study. The sample of participants included 20 sport parents (6 females and 14 males, 40–63 years of age) and 21 coaches (5 females and 16 males, 22–64 years of age) from youth competitive team sports (total **N* *= 41 participants).

Due to the COVID-19 pandemic, data collection was conducted via Zoom interviews. Data were collected between September 2020 to December 2020; during this time, youth athletes were participating in their respective sport[s] through virtual training and/or in-person training while adhering to public health guidelines for COVID-19. Semi-structured interviews [32] were conducted online at a time that was convenient for the participants, and all participants’ interviews were audio-recorded with the participants’ consent. All interviews were transcribed verbatim, personal identifiers were removed, and pseudonyms were used during transcription and interpretation of results to maintain anonymity. Parent interviews lasted 23–68 minutes (**M* *= 50 min, **Total* *= 986 min, 266 pages of transcription), while coach interviews ranged from 33 to 103 minutes (**M* *= 62 min, **Total* *= 1252 min, 329 pages of transcription).

### Data analysis

The analysis process followed Braun and Clarke’s [33] six phases of reflexive thematic analysis. The thematic analysis began with the researcher becoming familiar with the data, followed by generation of initial codes, and categorization of codes into potential themes. The researcher then reviewed the themes to ensure the themes operated in relation to the entire data set and created a thematic ‘map’ of the analysis. The analysis procedure concluded with defining the themes and production of the research report. The researcher viewed data saturation as a point at which he made a situated and interpretative judgement to stop coding and move to theme generation [34]. Thus, saturation was considered as information power which refers to the quality and relevancy of information provided by the participants rather than its quantity [34]. The researcher acknowledged his role in knowledge production and approached the data analysis with theoretical awareness and transparency [33]. Participants received a clear study description and were free to elaborate and provide additional information on various topics of discussion in interviews. Weekly meetings with the research supervisor provided critical feedback. The researcher maintained a reflexive journal to reflect on his philosophical assumptions and the impact on the research process [35]. Member reflection was also included to enhance credibility and allow participants to clarify their statements [36].

## Results

**Fig 1:**
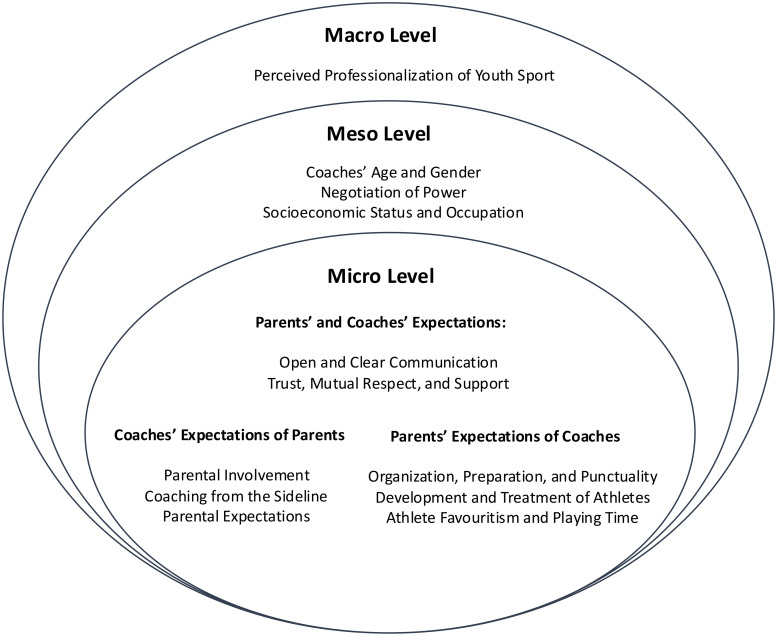
Micro, meso, and macro level factors influencing the coach-parent relationship. Legend: This figure illustrates a conceptual framework organizing key influences on the coach–parent relationship, derived from qualitative analysis, using Bronfenbrenner’s ecological systems theory. Micro-level factors reflect interpersonal dynamics between individual parents and coaches, including communication quality, trust, support, expectations, and behaviors. Meso-level factors describe broader social influences such as perceived gender roles, power negotiations within sport settings, and socioeconomic markers. Macro-level influences capture structural and systemic forces, including the commercialization and professionalization of youth sport.

See Fig 1 for a descriptive model of the current findings.

### Ingredients of a healthy relationship

Parents and coaches reported that *open and clear communication* greatly influenced their relationship. For instance, Simon [parent] said, “I think it just goes back to, again, communication, open communication and regular communication, using a variety of different means…always having that regular communication just to make sure that we know what’s expected of us.” In addition, parents and coaches reported that *being approachable and accessible* was an important factor in their relationships with one another. For example, Connor [coach] said “Being approachable…if the people know that they can send you that text or they can send you that email or pull you aside and say can I talk to you in person, I think that definitely goes a long way.”

Parents and coaches spoke about the importance of *honesty and transparency* in their communication. For example, one of the parents stated, “It’s all about transparency. When people don’t feel like their vote counts as much as other parents or, when they don’t feel like they’re being privy to information that other parents are, that’s going to chisel away at your culture.” Parents and coaches also highlighted the benefits of the *24-hour rule* for their day-to-day interactions. This rule refers to the mindful practice of waiting 24 hours before taking action [e.g., calling or sending an email] concerning something that happened. It’s the act of slowing down one’s thought process, and to allow adequate time to gather necessary information. Harry [coach] added “It gives me a chance to process my actions and take my notes into account so if we do have a conversation 24 hours later, I’ll be able to say, this is what happened here… I could’ve done this differently.”

Parents and coaches identified *trust and distrust* as a prominent characteristic of their relationship. For instance, Mateo [coach] said “Parents need to trust the coaches that they’ve paid…they’ve paid to play for me, they need to trust me, the moment that there’s no trust between myself and transparency between myself and that family, is where there is problems.” In contrast, Harry [coach] spoke about how distrust can lead to unhealthy parent-coach relationships. He said, “If your actions are against what you said you were going to do or there is distrust…any level of mistrust, or your actions not matching what you said you would provide, I think can cause friction.” Parents and coaches also thought showing *respect and support* towards one another would positively impact their relationships. For example, Carlie, a parent of a competitive female soccer player, said “I think showing the coaches that [parents] support them, that you respect the coach’s decision and their ability to coach. If parents do that, then I think that helps the coach, it empowers the coaches to run the team.”

### Parents’ expectations of coaches

Parents also spoke about the importance of coaches *being organized, prepared, and punctual*. For example, Carlie [parent] noted “This coach seems to really put a lot of effort into preparing a development plan or what he hopes for the team to learn, and [he] communicates that.” James [parent] spoke about the importance of coaches’ organization, and how that could potentially lead to less criticism from parents:

The most important thing is the coach has to be organized and they can’t be winging their practices, just showing up…because practice time is the most important time. If [the coach] isn’t organized the practice is going to be poor, your results on the ice or the field are going to be poor…So if a coach is organized and disciplined it’s very hard for a parent to complain.

Another coach-related feature that seemed to influence the coach-parent relationship was associated with *how coaches develop and treat their athletes*. For example, Chris who is a parent of a youth hockey player said, “When they come out of the rink, and you find out the coach has been yelling at the kids about what they did wrong, that’s when that [relationship] starts to erode quickly…that’s where things go sideways for me.” Furthermore, *perceived athlete favouritism and decisions about playing time* were also indicated as coach-related factors that can influence the relationship between parents and coaches. Jack [parent] mentioned:

One of the main things that made it unhealthy for us is when coaches pick kids who, you know, shouldn’t be on the team, based on either friends with the coach, with the parents or I’ve heard [the coaches] get a little something on the side from whoever they get the sum of money…so that makes it unhealthy when we look at certain players, who shouldn’t be there or even when you look at certain players, who shouldn’t be on the ice at certain times…those are the things that I find unhealthy.

### Coaches’ expectations of parents

There were various parent-related factors that also influenced the coach-parent relationship. Extent of *parental involvement*, for instance, was indicated as a contributor to the relationship. For example, Adrian [coach], spoke about the influence of ‘helicopter parents’ on coach-parent relationships: “Helicopter moms just wanting everything perfect for their kid, and when it doesn’t work out, they tend to lose their mind and then there’s obviously a reflection of that decision on the coach, on the training staff whoever you have.” Faith [parent] also said “You hear horror stories of parents…sending emails, cornering coaches, going into the dressing room. If I do those things, the coach will get frustrated with me and resentful and probably cut my kid.” This perception appeared to be consistent among parents:

The parents in competitive hockey they can be very nasty. The kids are no longer listening to the coaches; the coaches now, unfortunately, have taken a back seat and the parents are controlling the game. That’s just a foundation for an awful situation to continue to snowball. [Mackenzie, parent]

*Parental expectations* regarding their children’s sport participation [i.e., playing time] was another parent-related factor that seemed to affect parent-coach relationships in youth sport. For instance, Nathan, a parent, said, “Some people they think their kid is the best no matter what…so, if that person wants to be troublesome and thinks his kid should be on ice or field more than somebody else’s kid, I can see coaches getting frustrated.” Diego, a basketball coach, said, “Some parents have unrealistic expectations, and they expect their kid to play more than other kids and then they start being negative towards the coach or saying stuff behind the coach’s back; it gets back to the coach.”

### Coaches’ negotiation and use of power

Another factor that seemed to affect parent-coach relationships was the perceived power dynamic between parents, coaches, and athletes. For instance, Adrian [coach] said, “I’m gonna be honest with you, the teams that I’m involved, everybody is begging to get on these teams, like they’re top echelon teams, right? So, no parent would ever dare to say anything…nobody’s gonna fuck around.” Connor [coach] also said, “[Parents] have that voice to speak up. But at the end of the day, I’m the coach of the team and I get to make the final decision with my coaching staff as to which way we’re going to go.” Luca [coach] stated, “In the [Greater Toronto Hockey League] once you sign the card, [athletes are] basically the property of that team. You can’t leave that team because I would have the ability to make sure you don’t play for any other team.” Interpretations around the perceived power imbalance was also shared by parents. For example, Dalton [parent] said:

It’s a competitive team, the coach holds all the cards. Unless your kid is Wayne Gretzky, you don’t hold any cards, and how many parents have Wayne Gretzkys? So, team is going to have a roster of 19 kids, maybe two or three of those kids are really good and the rest of them are kind of ok and having fun. Those parents have no leverage, really…it’s competitive, the parent has no role, unless of course, they start paying more, which is a nice way of saying bribe right? It’s an unequal relationship, the parent has no say.

Likewise, Maryam [parent] added:

I feel like nobody ever wants to say anything because nobody wants to be the black sheep on the team. It’s a bit of a privilege to play at a higher level in hockey so maybe if your kid isn’t a superstar, like, I want to make sure he still plays there. So, you kind of sound very tentative, more tentative than I would with a teacher at school who, you know, I know the education system.

### Perceived professionalization of youth sport

The professionalization of youth sport refers to the cost of youth sport and financial proposals [offered by parents to coaches], and the prominent [if not exclusive] focus on performance outcomes that consequently transform the coach-parent relationship to a transactional relationship. For instance, Diego [coach] said:

My daughter plays rep soccer, it costs $4,000 dollars a year, right? So, there’s a lot of money involved. And when people are paying for stuff, they think they have a right to complain or say what their opinion is.” Likewise, Brian [parent] mentioned “As a parent, to invest $3000 dollars on A, or AA and then AAA in the [hockey league] can be as much as $12,000…if you’re not satisfied with the end result, you’re going to take your funding somewhere else right?

## Discussion

The purpose of this study was to explore parents’ and coaches’ perceptions of the factors that influence the coach-parent relationship in competitive youth team sports. At a more proximal level, participants highlighted factors related to expectations held of the other party such as transparency and honesty, and providing specific and performance-based feedback as important elements of their communication, which is similar to results from previous studies of coach-parent relationships in youth sport [13,16]. Our results also include parents’ expectations of coaches, and coaches’ expectations of parents that influenced the coach-parent relationship. While previous research has demonstrated the varied effects of parents’ expectations and involvement on youth athletes and coaches [16], our results also revealed that parents’ unrealistic expectations regarding their children’s playing time may be driven by various fees [i.e., ice rental, private training] that parents pay.

Another novel contribution from Study one concerned parents’ and coaches’ perceptions about coaches’ age and gender with respect to the coach-parent relationship, and the perceived power imbalance between coaches and parents. Furthermore, we identified the professionalization of youth sport as a contributor that has an over-arching influence on the coach-parent relationship. The professionalization of youth sport may have the effect of making athlete development a commodity by adopting a pay-to-play format; this can in turn situate parents as opinionated investors and consumers of their children’s sport participation [37,38]. Professionalization of youth sport may exacerbate economic marginalization and class disadvantages in organized competition [39].

While Study One offered an account of the key factors that shape coach-parent relationships, it became clear that these relationships are not static. Rather, participants described how their relationships evolved—sometimes strengthening through trust and collaboration, and at other times breaking down due to unmet expectations or conflict. However, no existing models adequately explain this developmental progression. This gap led directly to the design of Study Two, which sought to build on the insights from Study One by developing a grounded theory of how coach-parent relationships unfold over time in competitive youth team sport settings.

## Study Two

The purpose of Study two was to develop a conceptual model to explain how parents and coaches in organized competitive youth team sports develop their relationships with one another over time.

## Methods

To investigate the developmental trajectory of the coach-parent relationship, a constructivist grounded theory [29] approach was deemed an appropriate methodological choice for the present study. Given the exploratory aim of this research, and in the absence of a comprehensive pre-existing theoretical framework on coach-parent relationships, we used grounded theory methodology to inductively build theoretical understanding directly from participant experiences [29]. In grounded theory research, data collection and analysis occur simultaneously, and each informs and restructures the other [29]. Grounded theory is often used to examine interpersonal dynamics of groups, known as social processes [29].

### Participant recruitment and data collection

Upon receiving ethical approval from the University of Toronto Research Ethics Board [REB #39710], data collection began in an iterative fashion with subsequent sampling informed by the analysis at each stage of data collection. The researcher used purposeful sampling [40] to recruit parents and coaches of competitive youth sports teams [U10-U15] within a large urban centre in Ontario, Canada. Recruitment followed the same procedures as part one, although data collection was completed independently and with a new sample of participants. Parents and coaches were provided with information letters and consent forms and provided written consent prior to their participation in the study.

The first phase of participant recruitment included three parents and three coaches from organized competitive youth team sports. Subsequent recruitment of participants was informed by initial analyses and theoretical sampling [29], where the developing categories and the researcher’s increasing understanding of the theory directed the sampling of future participants. Based on the developing insights and concepts, the researcher conducted a delayed literature review [41] to gain a better understanding of how different components of the coach-parent relationship could be connected to one another. Following the first round of data collection and analysis, the researcher sought to recruit parents and coaches who had particular experiences in organized competitive youth team sports, or for whom certain concepts appeared important. The second phase of participant recruitment thus involved four parents and five coaches from organized competitive youth team sports. The ongoing analysis of the data highlighted the need to further recruit parents and coaches to explore the developing concepts such as ‘the influence of culture’, ‘generational differences’, ‘communication’, and ‘separation’ within the coach-parent relationship. Thus, the third phase of participant recruitment consisted of three parents and two coaches from organized competitive youth team sports. In total, 10 parents [9 females and 1 male] and 10 coaches [2 females and 8 males] from organized competitive youth team sports [Hockey, soccer, basketball, volleyball, and football] participated in the study.

Participants were recruited until the researcher and his critical friends believed that theoretical saturation had been reached [38]. In constructivist grounded theory the notion of theoretical saturation signifies that core categories developed from the research process are explained with adequate data to the extent that the incorporation of new data provides no additional insight [29]. Considering the interpretive approach within constructivist grounded theory, the researcher acknowledges that theoretical saturation is a subjective exercise and recognizes both the importance and limitations of this subjectivity [29]. This conceptualization aligns with grounded theory’s aim to develop an explanatory theory of the social phenomenon its context [38].

Semi-structured interviews [32] were conducted and all interviews were audio-recorded with the participants’ consent. To gain a better understanding of how parents and coaches develop their relationships, the interviews with parents and coaches were informed by a life story approach [42]. Parents and coaches were asked to create a ‘timeline’ of their involvement in competitive youth sports, and reflect on their relationships with coaches/parents along the way, and write down key events and/or memories in relation to each coach/parent. Additional questions were asked based on what the participants chose to discuss in their interviews. Informed by theoretical sampling at each stage of data collection, the interview guide was modified over the course of the study to allow for collection of data about topic areas that helped to develop concepts, uncover variations, and identify relationships between concepts [29]. For example, in the second round of interviews, questions were added to ask parents and coaches about different types of separation, and factors that informed parents satisfaction, which were theoretical concepts that were identified in the first round of analysis. Parent interviews lasted 30–60 minutes [*M =* 46 minutes, **Total* *= 464 minutes, 120 pages of transcription], while coach interviews ranged from 28 to 95 minutes [**M* *= 57 minutes, **Total* *= 579 minutes, 143 pages of transcription]. The interviews were transcribed verbatim, personal identifiers were removed, and pseudonyms were used during transcription and interpretation of results to maintain anonymity.

### Data analysis

Within constructivist grounded theory the iterative processes of data collection and analysis, along with active engagement between the researchers and research participants, co-construct the theory [29]. The researcher employed inductive and deductive analysis as needed, gathering additional data through abductive reasoning to explain unanswered or unexpected observations [29]. The coding process consisted of initial coding, focused coding, theoretical coding, memo-writing, and constant comparison [29].

Memo-writing was a crucial analytic strategy in developing the grounded theory because it encouraged the researcher to analyze the data and codes early in the research process [29]. Memos were written to summarize the researcher’s thoughts, capture the comparisons and connections that the researcher made, and provided more specific questions for the researcher to answer [29]. Likewise, constant comparative methods were used to compare data to data, incident to incident, and categories to categories [29].

## Results

**Fig 2:**
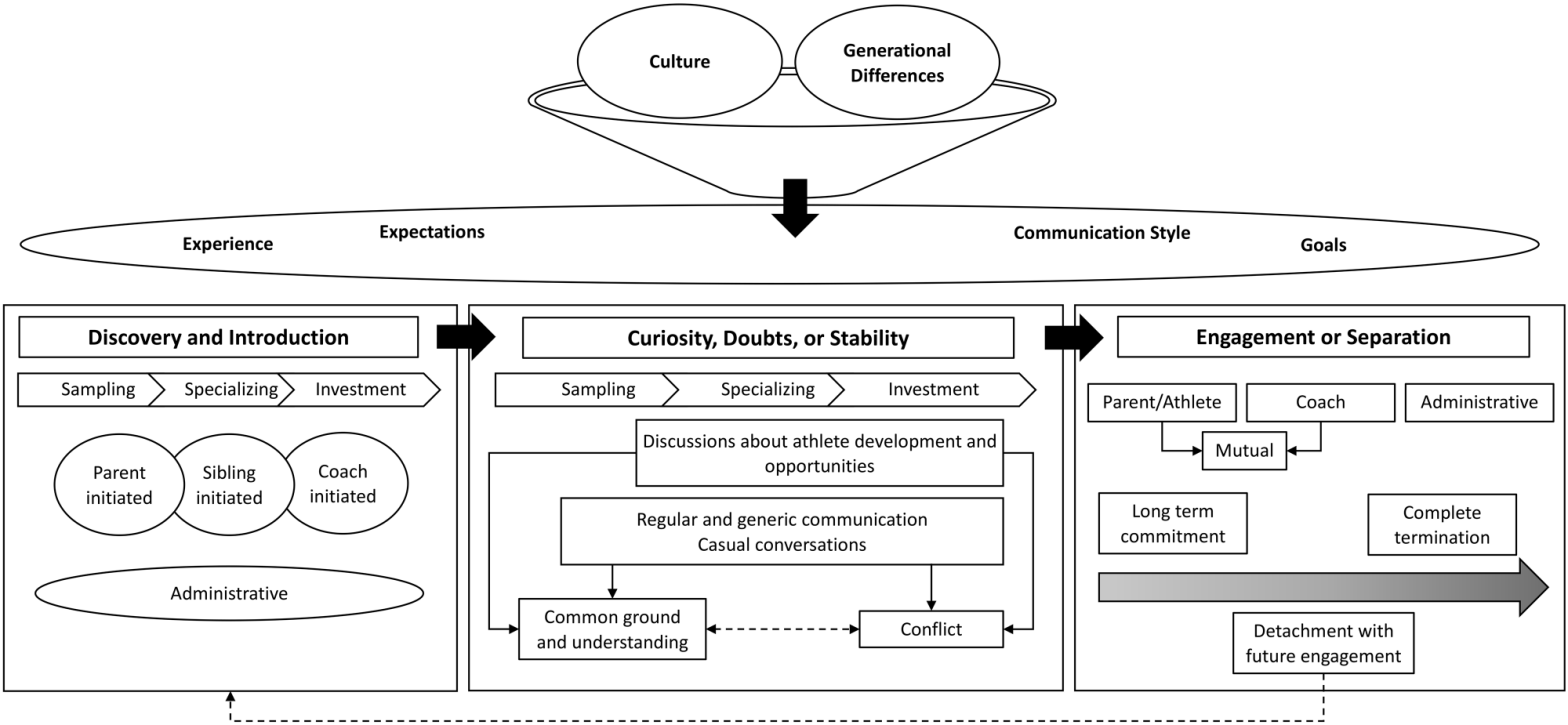
Conceptual model of coach-parent relationships in organized competitive youth team sports. Legend: This figure presents a conceptual model outlining the dynamic evolution of coach–parent relationships across three distinct phases: (1) *Discovery and Introduction*, (2) *Curiosity, Doubts, or Stability*, and (3) *Engagement or Separation*. The model is grounded in participants’ narratives and highlights how relational outcomes may vary based on interpersonal, contextual, and administrative factors. Within each phase, specific events such as recruitment, athlete development conversations, casual communication, conflict resolution, and separation decisions shape the relationship trajectory.

A conceptual model is first presented which outlines the developmental trajectory of the coach-parent relationship in organized competitive youth team sports [Fig 2], followed by explanation of key themes and aspects within the model.

### Overview of conceptual model

Based on participants’ experiences, the model comprises three sequential and dynamic phases: [a] *Discovery and introduction*, [b] *Curiosity, doubts, or stability*, and [c] *Engagement or separation*. These stages reflect how coach-parent relationships are formed, evolve, and ultimately stabilize, shift, or dissolve over time. Rather than occurring in a fixed or linear manner, movement between phases is influenced by various interpersonal and contextual factors. Across all phases, the model is shaped by broader sociocultural and structural forces, including generational differences, organizational policies, and prevailing norms surrounding sport professionalisation. Key influences such as expectations, communication styles, previous experiences, and relational trust play critical roles in determining whether a relationship progresses, stagnates, or terminates. The influence of culture and generational differences among parents were also included as overarching factors that influenced the coach-parent relationship. In addition, external influences such as expectations, experience, goals, and communication styles contributed to the development of the coach-parent relationship. The model provides a grounded and process-oriented understanding of coach-parent dynamics, offering insight into how these relationships can either support or undermine positive youth sport experiences. The remainder of this section unpacks each phase in detail, using participant quotes to illustrate the relational patterns and turning points that characterize the trajectory.

#### Phase one: Discovery and introduction.

The first phase of the coach-parent relationship was characterized by parents’ and coaches’ attempts to discover and find one another. This stage focuses on the process through which parents and coaches get introduced to one another. The process of introduction was divided into four entities based on the key agent[s] who initiated this process. For instance, ‘parent initiated’ refers to when a parent of an athlete reaches out to a coach to inquire about potential opportunities for their child to join a specific team or be trained by a particular coach. Likewise, ‘coach initiated’ refers to when a coach reaches out to a parent of an athlete to recruit the athlete for their team. ‘Sibling initiated’ refers to when a parent of an athlete chooses to sign up their younger child to be trained by the same coach [or in the same organization] as their older child. Lastly, ‘administrative’ refers to when club administrators choose to change the coach of a team [i.e., hiring a new coach, or moving the coach to a younger or older team], when the coach decides to retire or follow their child as they get older [i.e., parent coaches who choose to coach their child through different developmental stages], or when parents’ ability to select a new team/coach is limited by geographical boundaries put in place by sport organizations. Regarding parent-initiated relationships, Kyle [coach] talked about his experience with parents approaching him to inquire about tryouts for his team. He said” [The parent] is like ‘I really like what you’re doing.’ It was his choice. He came up to me…he is like ‘when are tryouts? My daughter is so-and-so and I want her to come try out for your team.’”

Regarding coach-initiated relationships, John [coach] described his experience of approaching an athlete’s parents to ask about their child and find out if they were interested in joining his program. He said “We realized that the kid was one of the better players out there. So, we approached them to ask them if Noah would like to play on our team.” Participants also discussed how the process of introduction and discovery between parents and coaches was often influenced by parents’ previous experiences with their younger child. Shannon [coach] described this process and said “I think it’s very rare that its coach initiated that early on. I think it’s more parent initiated, or sibling initiated. If another sibling in the family is played, a lot of times the other sibling wants to play.”

Furthermore, participants highlighted how sport administrators and organizational rules may influence the initiation stage of their relationship. Participants’ quotes in this regard describe parents’ and coaches’ lack of choice in selecting a coach or a team respectively. For instance, in relation to administrative initiation of coach-parent relationships, Tommy [coach] said, “Hockey in our area is different in that there’s residency rules on players, so [the parents] didn’t have a lot of options, it was either play for [name of the organization] or don’t play rep because of the residency requirements.”

#### Phase two: Curiosity, doubts, or stability.

The second phase of the coach-parent relationship was characterized by parents’ and coaches’ sense of curiosity and uncertainty, and pursuit of stability in the coach-parent relationship. This stage focused on the nature of interactions between parents and coaches, and possible outcomes related to such interactions. This phase consisted of two main types of discussions between parents and coaches related to: a] athlete development and opportunities, and b] regular and generic communication, or casual conversations between coaches and parents. Athlete-related discussions focused primarily on the progress the athlete was making in their respective sport [or lack of progress], and opportunities for the athlete to play their sport at a higher level [i.e., playing a year up, joining the provincial or national team program]. Regular and generic communication revolved around topics such as practice time and location, and weekly updates, whereas casual conversations focused on topics unrelated to sport such as family gatherings and everyday life events. In relation to discussions about athlete development and opportunities, Brook [coach] said, “We tend to have at least a big one-to-two-hour conversation at least once or twice a year specific to his daughter and what the goals are with her and how we’re going to try to achieve them.”

Regular and generic communication tended to focus on logistics. For example, Tommy [coach] stated, “I make a point of sending at least one weekly update to my families giving them as much information as we can…operational logistics information from, you know, this is how you order uniforms, sign up for hotels, for tournaments.” Casual conversations between parents and coaches appeared to concentrate on topics not related to sports. Brook added, “I also think when [parents] feel like I’m invested more in their personal life, too, I think it’s good like I’ll ask how their family is for example, you know it’s not just about soccer to me.” In summary, regular and generic communication, and casual conversations appeared to span across all three stages of development, whereas discussions about athlete development and opportunities tended to start towards the latter stages of the sampling phase.

Conversations between parents and coaches appeared to ultimately lead to common ground and understanding or conflict between the two parties. Participants discussed how they navigated such conversations; for instance, Kyle [coach] talked about his approach for making parents feel heard while at the same time explaining his philosophy to them: “[Parents] pay to be part of your program. So, I think listening, giving customers the opportunity to be heard I think is very powerful.” Likewise, Shannon [coach] described how she dealt with a parent who was frustrated about their child’s return to play timeline following an injury. She stated: “I told [the parent] that we talk the next day, I understood his frustration. Then we talked the next day. The next day he had cooled down, I had cooled down. He apologized. He understood that it’s a competitive situation.” Participants’ statements highlight the two-way nature of the coach-parent relationship. It was evident that to create a positive experience for parents and coaches, and to effectively solve potential conflicts, both parties needed to feel heard. In addition, Shannon’s account illustrated the importance of the 24-hour rule in the coach-parent relationship which indicates that parents and coaches [and sometimes athletes] should not discuss a game, or situations in a game until at least 24 hours after the fact.

Participants also discussed what factors could contribute or determine the outcome of conversations between parents and coaches, including communication style or expectations. For instance, Kyle [coach] discussed how parents’ preconceived notion about a conversation could affect the quality of that interaction: “Whenever I dealt with those negative parents, they came in already with their back up against the wall. They came in with the notion that you’re going to have an argument…all the negative parents come in with like guns blazing.” In relation to parents’ expectations, Savannah [parent] stated “We call it parent goggles. All of us have them where you think that your kid is better than they are. And then if a parent is coming from a different lens [than] the coach…then there will be a conflict.” In addition to communication style and experience, participants also discussed coaches’ over-promising and under-delivering as a factor that could affect the outcome of interactions between parents and coaches. Coaches’ promises tended to influence parents’ overall expectations. For example, Miriam [parent] said, “The problem is the approach of the coach. We’ve seen coaches that have sugar-coated what they think our kid can do. When you sugar coat and then you don’t actually give them that opportunity, that’s where that conflict comes from.”

#### Phase three: Engagement or separation.

The third phase of the coach-parent relationship was characterized by the level of engagement and commitment that parents and coaches displayed towards one another. Within this stage, parents’ and coaches’ level of engagement exists along a continuum ranging from ‘long-term commitment’, to ‘detachment with future engagement’, and ‘complete termination’. Within the conceptual model, long-term commitment refers to instances where parents and coaches established a long-standing relationship with one another. Detachment with future engagement refers to instances where parents and their child athlete decide to join a new organization [with new coaches], but later – perhaps after a season or two – choose to return to their previous organization and coaches. Development of the coach-parent relationship can therefore potentially support re-engagement later on for athletes who may disengage from sport [43,44]. Lastly, complete termination refers to instances where parents and coaches decide to part company, with no plans to maintain a relationship or re-connect in the future. Levels of engagement in the coach-parent relationship can be influenced by the parent [and their child], the coach, both the parent and the coach [mutual], or sport administrators and organizations [administrative]. For example, in relation to ‘detachment with future engagement’ Marcel [coach] discussed how parents sometimes chose to leave an organization, but later came back to the same organization. He said “At times [parents] don’t trust you or the organization that you’re in. They feel that the grass is greener…the grass is always greener on the other side. So, there is that detachment where they’ll leave and then they’ll come back.” In relation to mutual separation Kyle [coach] added, “[The parent and the player] need a new environment…it’s not a fault on me…they need more. So, if you can’t give them more then no worries, they go.” It appeared that mutual separation between parents and coaches occurred when both parties, and coaches in particular, maintained their focus on what is in the best interest of the athlete and did not view the situation as an attempt to delegitimize their program.

The relationship between parents and coaches often came to an end due to the coach retiring or taking a sabbatical from coaching. Within the conceptual model this type of separation is shown as administrative. Tara [parent] described her experience with administrative separation as follow: “Last year’s coach, he decided to retire from coaching. So we got a new a new coach at the AA level.” Similarly, Audrey [parent] said “He’s no longer coaching. He’s decided to take a break from coaching. But we are still great friends with him. I have a lot of respect for him as an individual. Our families are close.” The process of separation was often initiated by parents [in partnership with their children]. This is where parents and their child athlete decided to join a new program and work with a new coach; Brook [coach] spoke about how her relationship with an athlete and her parents was terminated by the parents, saying “[The parent] didn’t even tell me that his daughter was looking or that they were unhappy…they just kind of came to me the day that they had already committed to a different club to tell me that she was leaving.”

### Culture and generational differences

Participants discussed the influence of culture and generational differences among parents on the coach-parent relationship. Culture referred to parents’ and coaches’ ethnicities, their childhood experiences, and the society in which they were raised and how those dimensions shaped the way they think and behave. Parents’ and coaches’ cultural background also appeared to influence their expectations of one another, and whether they viewed certain behaviours as acceptable or unacceptable. For instance, Jade [parent] discussed how her upbringing and ethnicity shaped her expectations. She said, “I grew up in a Caribbean household…the approach has always been kind of an old school approach. Whenever I looked for coaches for my daughter, I always looked for that approach because I know that she is accustomed to that.”

Furthermore, generational differences among parents seemed to affect how they viewed coaches’ actions towards their children. It seemed that parents of older generations [i.e., born in mid-to-late 1970s] displayed a higher tolerance for behaviours that were deemed by younger generation parents as unacceptable. Miriam [Parent] described her thoughts on generational differences among parents and how that influences parents’ expectations. She stated:

I want to say that I’m maybe on the cusp of this generational difference, I’m forty-five almost. But I think older than me, parents tolerate a little bit more in terms of what coaches do, and I feel like people that are younger than me think that their kids are like beautiful, wonderful flowers that can’t be touched or broken.

## General discussion

Despite recent studies in this area that have addressed different aspects of the coach-parent relationship in youth sports, the current studies provide additional information regarding parents’ and coaches’ perceptions about the potential influence of factors such as age and gender, socioeconomic status and occupation, and professionalization of youth sport on the coach-parent relationship. Furthermore, the current research adds critical information about the development of coach-parent relationships in youth team sports contexts, and how parents and coaches conceptualize and experience different stages of this relationship [45].

Relationships between parents and coaches in youth sport are generally positive but can involve conflict, with early research framing parents along a continuum of involvement that often casts them as problematic [13,46]. Recent studies have shifted focus toward characterizing these relationships and promoting strategies for collaboration to enhance harmony and reduce conflict [22,28,47]. The present studies contribute to existing research in this area by supporting previous findings around the importance of trust and support within the coach-parent relationship. More specifically, results from Study one demonstrated that parents’ trust is often developed through coaches’ treatment of their athletes, transparent communication, and actions that support such communication. Furthermore, results from Study one draw attention to the power dynamic between parents and coaches in youth sport, with parents having less power than coaches. While the notion of power had been noted in previous research [13], the present findings describe mechanisms that may contribute to the development of power imbalance between parents and coaches. Furthermore, the results from the present studies also demonstrated that parents’ perceptions of female coaches were influenced by traditional gender norms, which may consequently discourage female coaches’ continuous involvement in youth sport coaching [43]. In addition, results revealed that parents’ view and acceptance of certain coaching practices may be influenced by their cultural background [44]. Therefore, future researchers and sport administrators should not adopt a ‘one size fit all’ approach when it comes to resolving conflict between parents and coaches and should account for parents’ and coaches’ previous experiences and social background.

The developed model illustrates a three-stage process through which parents and coaches develop their relationship with one another. This conceptual model makes a contribution to the literature in that it: is grounded in stakeholders’ experiences; demonstrates the relationship between personal, social, and organizational factors; illustrates the developmental trajectory of the coach-parent relationship through three stages; and builds the foundation for future researchers to focus their research on investigating specific stages of this relationship rather than examining the coach-parent relationship as one entity. Although developed inductively, the model resonates with recent theoretical work on parental involvement in youth sport [27] particularly regarding how misaligned expectations and unclear boundaries can strain coach-parent dynamics. Importantly, the model can also be understood within the broader coach-athlete-parent triad framework [CAP; 20], which emphasizes the interdependence and relational dynamics among all three parties. While much prior research on the CAP triad has examined these relationships at a single point in time, the current model uniquely contributes a developmental lens by illustrating how the coach-parent dyad evolves and how its trajectory can influence the overall functioning of the triad. For instance, moments of relational strain or trust-building between coaches and parents may directly affect athlete outcomes, especially when communication or values across the triad are misaligned. Our findings also highlight how external pressures, such as sport professionalization and organizational policies, can introduce systemic constraints that ripple through the triad, influencing how coaches and parents engage with each other and with the athlete. Thus, this model not only advances conceptual understanding of the coach-parent relationship but also offers a foundation for examining how changes within this dyad may impact broader athlete development and wellbeing within the triadic context.

In addition, results from Study two highlight the influence of factors such as culture and generational differences among parents on the development of the coach-parent relationship. Collectively, this research answers previous researchers’ call to consider the influence of factors such as age, gender, culture, and previous experiences on the coach-parent relationship [13]. Previous research has highlighted the association between one’s social and cultural backgrounds (i.e., past experiences, internalized norms, and social environment]) and their expectations [22,44]. Future research should investigate the coach-parent relationship among diverse populations such as recreational sports, BIPOC [Black, Indigenous, and Peoples of Colour], and single parent families.

### Coach-parent relationships and the professionalization of youth sport

The professionalization of youth sport emphasizes competition, winning, and talent development, with expectations for polished skill displays and a focus on statistics and outcomes, mirroring professional sports [48,49]. This trend has been linked to negative consequences, including limited time for unstructured play, injuries, prolonged recovery times, and reduced access for marginalized children, such as those with disabilities and children of color [49,50]. Within the present studies, the results illustrated the impact of professionalization of youth sport on the coach-parent relationship. In Study One, a prominent emphasis on performance outcomes appeared to shift the coach-parent relationship away from mutual support and collaboration toward a transactional, consumer-service model. This reframing alters the nature of communication between coaches and parents, increasing pressure on coaches to justify playing time, training decisions, or team selection in terms of value and return on investment. It may also reduce opportunities for genuine relationship-building, as conversations become more outcome-focused and transactional. In some cases, this shift created conflict or dissatisfaction, particularly when expectations were unmet or when coaches perceived their expertise as being challenged. These findings support Dorsch et al.’s [50] conceptualization of the youth sport environment as one where systemic structures shape individual roles and expectations. Our data suggest that the growing commercialization of youth sport not only alters athlete experiences but also complicates the relational fabric between the key adults tasked with supporting them.

### Applied implications

These findings provide several implications for sport organizations and stakeholders in youth sport to support coach-parent relationships. In fact, the current study compliments recent work by Eckardt & Dorsch [51] wherein they highlight the importance of fostering cooperation among parents, coaches, and administrators in professional youth soccer academies. They found that cooperation often follows a trial-and-error approach due to a lack of evidence-based guidance, leaving parents and coaches to navigate their roles through experiential learning or observing others. The current findings shed light the value of educating coaches and parents about each stage of the relationship, events within each stage, and strategies for specific situations can be valuable. Using the conceptual model produced in this research can help coaches and parents navigate their relationship by understanding what to expect in each stage, leading to more understanding and potentially less conflict. This knowledge translation strategy can be particularly beneficial for new sport parents without prior experience, who may face challenges and tension [52].

Our results also emphasize the need for sport organizations to establish opportunities for female coaches to receive adequate support for navigating challenging relationships with specific parents [53,54]. Sport organizations should create a safe and supportive environment for their female coaches to engage in productive conversations with parents to voice their concerns and provide their parent group with research-based information about the involvement of female coaches in youth sport. In addition, sport organizations can further support their coaches by proactively identifying and managing problematic behaviours exhibited by sport parents, thereby alleviating the burden on female and male coaches to address these challenges independently.

### Limitations and future research

Considerations regarding the limitations of these studies include participant recruitment focusing on English speakers in a specific urban area; future research can contribute to this body of research by recruiting from diverse cultural and socio-economic backgrounds. Additionally, understanding the experiences of under-represented groups like single parents and the LGBTQ community is important when exploring parent-coach relationships. Similarly, as Santos et al. [14] noted, more research on coach-parent relationships is needed within sport contexts that include younger athletes (i.e., 6–9 years old). Further, this study focused on coach-parent relationships in competitive sports, therefore, we did not capture the experiences of parents and coaches in recreational sports. Future research should explore coach-parent relationships across different competition levels. Similarly, the perspectives of athletes were not included, as the study design centered on the dyadic coach-parent relationship. However, we recognize athletes as key stakeholders and encourage future work to triangulate perspectives across athletes, parents, and coaches.

Another limitation was the exclusion of athletes’ perspective on the coach-parent relationship, which should be considered in future studies. Virtual data collection and the impact of COVID-19 social distancing measures at the time of the research may have influenced the interview process and parents’ and coaches’ perceptions of their relationship. While this format enabled broad geographic reach and scheduling flexibility, it may have influenced the depth and nuance of participant responses [55]. Building rapport through a screen can be more challenging than in-person settings, and limited access to non-verbal cues may have affected the interpretation and flow of dialogue. Despite these constraints, the interviewer employed active listening techniques, verbal affirmations, and flexible questioning to encourage openness and engagement. Nonetheless, the potential impact of the online format on disclosure and relational depth is acknowledged as a contextual limitation of the study. In addition, the research did not adopt a longitudinal approach to explore participants’ experiences over time, representing an important area for future research. Future work can build upon the findings here by following parents and coaches over the course of a season to examine how their experiences align with the proposed grounded theory.

Recent calls have been made to consider more transdisciplinary interpretation of coach-parent relationship research by applying more diverse conceptual models [14]. The present work’s conceptual model enables future researchers to focus on specific stages of the coach-parent relationship rather than treating it as a singular entity. Future studies should also explore how wider discourses, such as historical and cultural influences, impact each stage of the coach-parent relationship [22]. For example, researchers can investigate how parents’ cultural backgrounds influence their decision-making during the discovery and introduction stage, as well as their selection process for a coach. Furthermore, examining the extent of common understanding and conflict among coaches and parents with similar and diverse cultural backgrounds could provide valuable insights.
